# Identification of archaeal proteins that affect the exosome function in vitro

**DOI:** 10.1186/1471-2091-11-22

**Published:** 2010-05-27

**Authors:** Juliana S Luz, Celso RR Ramos, Márcia CT Santos, Patricia P Coltri, Fernando L Palhano, Debora Foguel, Nilson IT Zanchin, Carla C Oliveira

**Affiliations:** 1 Department of Biochemistry, Institute of Chemistry, University of São Paulo, 05508-000, São Paulo, SP, Brazil; 2 Center for Structural Molecular Biology, Brazilian Synchrotron Light Laboratory, LNLS, 13083-970, Campinas, SP, Brazil; 3 Institute of Medical Biochemistry, CCS, Federal University of Rio de Janeiro, 21941-590, Rio de Janeiro, RJ, Brazil; 4 Department of Helminthology, Fiocruz, 21045-900, Rio de Janeiro, RJ, Brazil; 5 Department of Molecular, Cell and Developmental Biology and Center for Molecular Biology of RNA, University of California, Santa Cruz, California 95064, USA

## Abstract

**Background:**

The archaeal exosome is formed by a hexameric RNase PH ring and three RNA binding subunits and has been shown to bind and degrade RNA *in vitro*. Despite extensive studies on the eukaryotic exosome and on the proteins interacting with this complex, little information is yet available on the identification and function of archaeal exosome regulatory factors.

**Results:**

Here, we show that the proteins PaSBDS and PaNip7, which bind preferentially to poly-A and AU-rich RNAs, respectively, affect the *Pyrococcus abyssi *exosome activity *in vitro*. PaSBDS inhibits slightly degradation of a poly-rA substrate, while PaNip7 strongly inhibits the degradation of poly-A and poly-AU by the exosome. The exosome inhibition by PaNip7 appears to depend at least partially on its interaction with RNA, since mutants of PaNip7 that no longer bind RNA, inhibit the exosome less strongly. We also show that FITC-labeled PaNip7 associates with the exosome in the absence of substrate RNA.

**Conclusions:**

Given the high structural homology between the archaeal and eukaryotic proteins, the effect of archaeal Nip7 and SBDS on the exosome provides a model for an evolutionarily conserved exosome control mechanism.

## Background

The exosome is a 3'-5' exonucleolytic multisubunit complex found in archaea and eukaryotes [1-6]. In yeast, where it was first described, the cytoplasmic exosome comprises ten subunits, whereas the nuclear exosome is formed by eleven subunits [7]. Eight subunits show sequence similarity to exoribonucleases, and for some of them catalytic activity was demonstrated *in vitro *[1,8]. Recent data indicate, however, that only the subunits Rrp6p and Rrp44p have intrinsic catalytic activity [9]. In yeast, depletion of individual exosome subunits leads to similar phenotypes [1,10-12], indicating that only the intact complex is functional *in vivo*.

Analyses of intra-complex interactions have provided the initial information on the yeast exosome architecture [12-15]. A working model for its structure and composition has been proposed based on mass spectrometry analyses of complexes purified under different conditions and on yeast two-hybrid interaction analyses [16,17]. According to this model, the RNA binding subunits (Rrp4p, Rrp40p and Csl4p) bind to one side of the RNase PH ring, which is formed by Rrp41p-Rrp45p-Rrp46p-Rrp43p-Mtr3p-Rrp42p [16], whereas the hydrolytic RNases (Rrp44p and Rrp6p) bind to the opposite side of the ring, although Rrp44p, the largest exosome subunit, may also interact with Rrp4p [16,17]. The crystal structure of the core human exosome has been recently determined, confirming the predictions for the structure of the eukaryotic complex [9].

The archaeal exosome is composed of four different subunits, two RNA binding subunits, aCsl4 and aRrp4, and two subunits containing RNase PH domains, aRrp41 and aRrp42 [4,6]. Three copies of an aRrp41-aRrp42 heterodimer form the RNase PH ring [18-22], which associates with three molecules of aRrp4 and/or aCsl4 [18,21]. The RNase PH ring has three active sites, responsible for the phosphorolytic RNase activity of the complex, formed in the interface between aRrp41 and aRrp42 [18-22]. aCsl4 and aRrp4 may have a flexible structure, which allows them to interact with RNA and bring it to the central pore of the RNase PH ring [21] and, subsequently, to the active site. Despite having three active sites, the narrow entry pore of the exosome allows the passage of only one single-stranded RNA molecule [22,23].

In eukaryotes, the exosome associates with specific protein cofactors in both cellular compartments. In the nucleus, the exosome has been shown to interact with Rrp47p, Mtr4p, the TRAMP complex, and Nop53p, and in the cytoplasm, with the Ski complex [[24-28], reviewed in [29]]. Rrp47p has been proposed to be a nuclear exosome cofactor required for the processing of stable RNAs [27]. Nop53p is a nucleolar protein that activates the exosome during processing of pre-rRNA 7S to mature 5.8S rRNA [28]. Mtr4p is an RNA helicase and is a subunit of the TRAMP complex, which is involved in RNA polyadenylation, directing the RNA for degradation by the exosome [29-31]. The Ski complex also contains a subunit with helicase activity, Ski2p, and is associated with the exosome in mRNA degradation [32,33]. The different protein cofactors that associate with the exosome may regulate its function.

Although the archaeal exosome has been shown to associate with other proteins [34], little is known about archaeal exosome cofactors or regulatory proteins. There are, however, some plausible candidates for exosome regulatory factors. Among them, PaSBDS, a protein encoded by a gene found in the *Pyrococcus abyssi *exosome operon, of which eukaryotic orthologues (Sdo1p in yeast, SBDS in human and in *Trypanosoma*) have been shown to be involved in ribosome maturation [4,35-38]. PaNip7 has been shown to bind RNA *in vitro *[39] and its yeast orthologue, Nip7p, is involved in rRNA processing and interacts with the yeast exosome subunit Rrp43p [40], corroborating the hypothesis of PaNip7 being a Pa-exosome cofactor. Pa1135 is a protein of unknown function, encoded by a gene found in the same operon as the RNase P subunit Rrp30 [4], and may also be involved in RNA processing. In this work, we show that PaSBDS and PaNip7 affect the exosome activity. PaSBDS competes with the exosome for binding to poly-A RNA, thereby slightly inhibiting its degradation by the complex. PaNip7 binds preferentially to U- and AU-rich RNAs, and strongly inhibits the exosome due to its association with both the exosome complex and the substrate RNA. Pa1135 on the other hand, though also binding U- and AU-rich RNAs in vitro, does not inhibit RNA degradation by the exosome. These findings indicate that PaNip7p and PaSBDS may be exosome regulatory factors. This is the first example of archaeal proteins regulating the exosome by inhibition.

## Results

### Pa-exosome complexes show different affinities for binding to oligo-RNA in vitro

**Figure 1:**
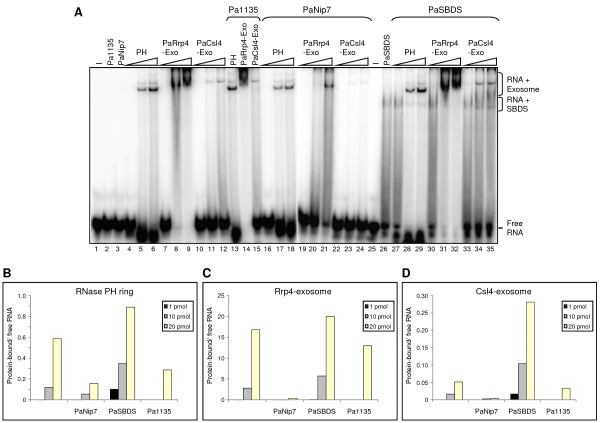
**Influence of putative cofactors on Pa-exosome poly-A RNA binding**. (**A**) Electrophoretic mobility shift assays with radiolabeled 14-mer poly-rA probe incubated with fixed amounts of the indicated purified proteins (100 pmol of either Pa1135 or PaNip7, or 200 pmol of PaSBDS), and increasing amounts of the exosome complexes (1, 10, or 20 pmol of either RNase PH ring, PaRrp4-exosome, or PaCsl4-exosome). Lanes 13-15, 20 pmol of exosome complexes. Proteins were incubated with 1 pmol RNA at 37°C for 30 min, and RNA-protein complexes were fractionated on 8% native polyacrylamide gels and visualized by phosphorimaging. -, No protein added to the reaction. Bands corresponding to free RNA oligo and protein-bound RNAs are indicated on the right-hand side. (**B-D**) Quantitation of bands visualized in RNA binding assay. The ratio of protein-bound RNA over free RNA in each lane was calculated for the three concentrations of exosome complexes used (1, 10, or 20 pmol), in absence or presence of 100 pmol PaNip7, 200 pmol PaSBDS, or 100 pmol Pa1135. (**B**) Effect of the three tested proteins on RNase PH ring exosome complex. (**C**) Effect of the proteins on the PaRrp4-exosome. (**D**) Effect on the PaCsl4-exosome.

The archaeal exosome complex is composed of the RNase PH ring (containing three aRrp41-aRrp42 heterodimers) and three molecules of the RNA binding subunits aRrp4 (aRrp4-exosome), or aCsl4 (aCsl4-exosome), or a mixture of both. The archaeal exosome has been shown to bind poly-A RNA, when incubated in the absence of phosphate [18,21]. Interestingly, the RNase PH ring subcomplex (apo-exosome) is able to bind RNA by itself in the absence of the RNA binding subunits [18,21]. We had previously reconstituted the *Pyrococcus *exosome *in vitro *[21], by assembling PhRrp41 and PhRrp42 (from *P. horikoshii*) and PaRrp4p (from *P. abyssi*). In this work, the same approach was used to reconstitute the *P. abyssi *exosome, containing PaRrp41, PaRrp42, and either PaRrp4 or PaCsl4. We first compared the RNA binding ability of the *in vitro *assembled *P. abyssi *apo-exosome, here designated as RNase PH ring, and the holo-exosomes PaRrp4-exosome, and PaCsl4-exosome through EMSA. The results show that upon addition of increasing amounts of the exosome complexes (1, 10 or 20 pmol) to a poly-A RNA, the free RNA band decreases, while bands appear in the upper part of the gel, which correspond to RNP complexes (Fig. 1A). The PaRrp4-exosome binds poly-rA three-fold more efficiently than the RNase PH ring, and nine-fold more efficiently than the PaCsl4-exosome (Fig. 1A, lanes 4-12). This difference in RNA affinity is probably due to the presence of the two RNA binding domains S1 and KH in PaRrp4, and confirms our previous observation of more efficient RNA binding by the *Pyrococcus horikoshii-abysii *assembled complex [21]. Interestingly, although PaCsl4 contains the RNA binding domains S1 domain and Zn-ribbon, we observed a less efficient binding of the PaCsl4-exosome to RNA than the apo-exosome (Fig. 1A; compare lanes 4-6 to 10-12). Incubation of the RNase PH ring with poly-rA resulted in the formation of an RNA-protein complex (Fig. 1A), but also in some degradation of the RNA (Fig. 1A; lanes 4-6). Since no phosphate was added to these reactions, degradation products might be due to residual phosphate present in the protein fractions. It is unlikely that this RNase activity is due to contaminant *E. coli *RNases co-purified with the exosome RNase PH ring, because *E. coli *extracts were incubated at 80°C for 30 minutes immediately before purification of the archaeal proteins. Similar residual phosphorolytic activities were reported for *Sulfolobus solfataricus *RNase PH ring [41]. Incubation of the PaRrp4-exosome with poly-rA resulted in the formation of RNA-exosome complexes of different sizes, resulting in the detection of RNA shifted bands and to some complexes that were retained in the gel slot (Fig. 1A). These larger complexes might result from binding of various exosome molecules to the same RNA, or from protein precipitation at higher concentrations, and were not included in the quantitative analyses of the experiments (Fig. 1B-D). These assays were performed at least three times, with different protein preparations and probes, and here a representative experiment is shown.

Although the exosome of several archaeal species have already been characterized [18-23,41], little is known of the protein factors that might interact with the Pa-exosome and regulate its function. In order to obtain insights into the association of the archaeal exosome with other proteins that are expected to function in RNA processing, a series of experiments were performed with three candidate proteins, PaSBDS, PaNip7, and Pa1135. PaSBDS does not bind poly-rC, binds poly-rU with low affinity, binds 10 nucleotides long poly-rA, but more efficiently longer poly-rA, and binds poly-rAU RNAs (Additional file 1 Figure S1A). PaNip7, has already been shown to bind RNA *in vitro *with higher affinity for U-rich RNAs [39], and here we show that PaNip7 also binds a poly-AU RNA (Additional file 1 Figure S1B) that contains complementary sequence and can form both intra- and inter-strand base-pairs. Similar to PaNip7 and PaSBDS, Pa1135 binds poly-AU RNA very efficiently, but differently from PaNip7 and PaSBDS, it does not bind to any of the RNA homopolymers tested (Additional file 1 Figure S1C).

### Effects of *P. abyssi *proteins on the exosome-RNA interaction

Consistent with the results described above, parallel EMSA with Pa1135, PaNip7 and PaSBDS and a poly-rA substrate showed that a band shift can be detected only for PaSBDS (Fig. 1A, lanes 2, 3, and 26, respectively). Incubation of the exosome complexes with RNA in the presence of these proteins revealed their effect on the exosome RNA binding activity. As shown here, although Pa1135 does not bind poly-rA, it inhibits 1.6-fold poly-rA binding by RNase PH ring and 1.7-fold binding by PaCsl4-exosome (Fig. 1B, D). However, Pa1135 does not show a pronounced effect on the PaRrp4-exosome binding to poly-rA (Fig. 1A, lanes 13-15; Fig. 1C). Since the exosome has a much higher affinity for binding to poly-rA than PaNip7, in a direct competition assay the exosome was expected to prevail over PaNip7 for poly-rA binding. Instead, the results show a strong decrease in the intensity of the bands shifted by the exosome in the presence of PaNip7 (Fig. 1A, lanes 16-24). PaNip7 caused a three-fold decrease in poly-rA binding by the RNase PH ring, a 40-fold decrease in binding by the PaRrp4-exosome, and a 20-fold decrease in binding by the PaCsl4-exosome. This result indicates that the decrease in the exosome binding to poly-rA caused by PaNip7 may be due to a direct interaction between PaNip7 and the exosome and not to direct competition for binding to poly-rA.

PaSBDS, on the other hand, binds poly-rA, and was expected to compete with the exosome for binding to this RNA. When PaSBDS and the exosome complexes are incubated with poly-rA, the band corresponding to the free RNA decreases in intensity and bands corresponding to PaSBDS-RNA and exosome-RNA become visible (Fig 1A, lanes 27-35). Poly-rA binding by PaRrp4-exosome was little affected by the presence of PaSBDS, indicating that PaRrp4-exosome has higher affinity for poly-rA than PaSBDS (Fig. 1A, lanes 30-32; Fig. 1C). The observation that PaSBDS-RNA complex results in a smear, instead of a well defined band, also indicates a low stability complex. Surprisingly, however, RNase PH ring and PaCsl4-exosome bound poly-rA more efficiently upon addition of PaSBDS (Fig. 1A, lanes 27-29 and 33-35, respectively). In summary, PaSBDS binds poly-rA and increases RNA binding by the RNase PH ring and the PaCsl4-exosome, but does not strongly affect the PaRrp4-exosome. Pa1135 and PaNip7, do not bind poly-rA with high affinity, but affect the exosome. Pa1135 affects RNA binding by the exosome complexes with lower RNA affinity, RNase PH ring and PaCsl4-exosome, while not affecting the PaRrp4-exosome, which has higher affinity for RNA. PaNip7 inhibits all the exosome complexes, and more strongly PaRrp4-exosome and PaCsl4-exosome.

### PaNip7 and PaSBDS interfere with exosome RNA degradation

**Figure 2:**
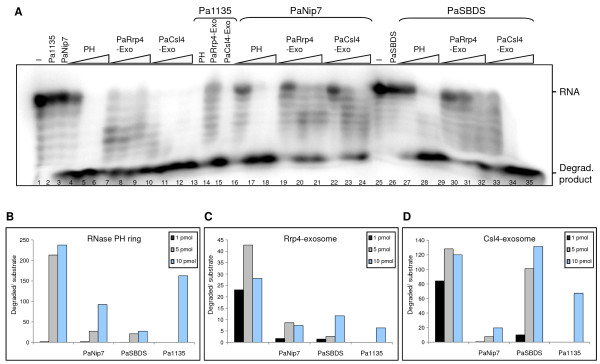
**Effect of the putative cofactors on Pa-exosome poly-A RNA degradation activity**. (**A**) RNase activity reactions were performed with 1 pmol radiolabeled 14-mer poly-rA probe incubated with fixed amounts of the indicated purified proteins (100 pmol of either Pa1135 or PaNip7, or 200 pmol of PaSBDS), and varying amounts of the exosome complexes (1, 5 or 10 pmol of either RNase PH ring, PaRrp4-exosome, or PaCsl4-exosome). Lanes 13-15, 10 pmol of exosome complexes. Proteins were incubated with RNA at 65°C for 15 min, in the presence of 10 mM NaH_2_PO_4_. Samples were separated on 8% denaturing polyacrylamide gel and visualized by phosphorimaging. -, No protein added to the reaction. RNA oligo and degradation products are indicated on the right-hand side. (**B-D**) Quantitation of bands visualized on denaturing polyacrylamide gels after RNA degradation assay. The ratio of degradation products over substrate RNA in each lane was calculated for the three concentrations of exosome complexes used (1, 5, or 10 pmol), in absence or presence of 100 pmol PaNip7, 200 pmol PaSBDS, or 100 pmol Pa1135. (**B**) Effect of the three tested proteins on RNase PH ring exosome complex. (**C**) Effect of the proteins on the PaRrp4-exosome. (**D**) Effect on the PaCsl4-exosome.

The archaeal exosome has been shown to degrade RNA *in vitro *[18,20,21], and based on the effect of PaNip7, PaSBDS, or Pa1135 on the exosome RNA binding, we decided to investigate whether the RNase activity of the complex was also influenced by these proteins. RNA degradation assays were performed in the presence of Tris-50 buffer and 10 mM NaH_2_PO_4 _(Fig. 2). Upon addition of 100 pmol Pa1135 to the reaction, little effect was detected on the degradation of a 14-nucleotide poly-rA substrate by RNase PH ring and PaCsl4-exosome, and higher inhibition was detected on PaRrp4-exosome (Fig. 2A, lanes 13-15; Fig. 2B-D). 100 pmol PaNip7, on the other hand, inhibited the RNase activity of all complexes, showing a stronger inhibitory effect on the holo-exosome complexes, PaRrp4-exosome and PaCsl4-exosome (3.8- and 6.1-fold inhibition of the RNase activity, respectively) (Fig. 2A; compare lanes 4-12 to lanes 16-24). Addition of 200 pmol PaSBDS inhibited the RNase activity of the RNase PH ring and PaRrp4-exosome up to eight-fold (mainly in the reactions containing lower amounts of these exosome complexes), but showed little effect on the PaCsl4-exosome (Fig. 2A, lanes 27-35).

**Figure 3:**
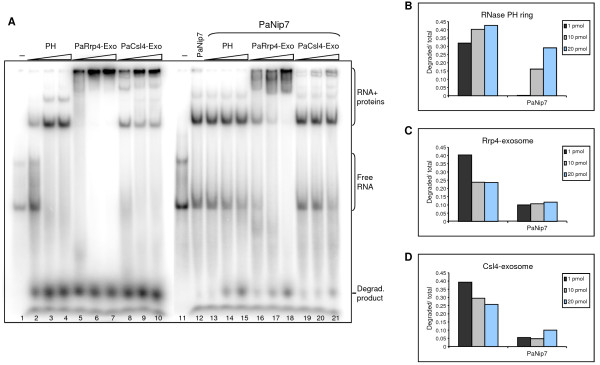
**Influence of PaNip7 on Pa-exosome RNA binding and degradation of poly-AU RNA**. (**A**) RNase protection assay with 1 pmol radiolabeled 21-mer poly-rAU probe incubated with varying amounts of the exosome complexes (1, 10, or 20 pmol of either RNase PH ring, PaRrp4-exosome, or PaCsl4-exosome), in the absence or presence of 100 pmol PaNip7. RNA was incubated with either PaNip7 or buffer at 37°C for 30 min, followed by the addition of the exosome complexes and further incubation at 65°C for 15 min. RNA-protein complexes were fractionated on 8% native polyacrylamide gels and visualized by phosphorimaging. -, No protein added to the reaction. Bands corresponding to free RNA oligo, protein-RNA complexes, and RNA degradation products are indicated on the right-hand side. (**B-D**) Quantitation of bands visualized on native polyacrylamide gels after RNase protection assay. The ratio of degradation product over total RNA in each lane was calculated for the three concentrations of exosome complexes used (1, 10, or 20 pmol), in absence or presence of 100 pmol PaNip7. (**B**) Effect of PaNip7 on the RNA degradation by the RNase PH ring exosome complex. (**C**) Effect of PaNip7 on the PaRrp4-exosome. (**D**) Effect of PaNip7 on the PaCsl4-exosome.

Since PaNip7 showed the highest inhibitory effect on the exosome poly-rA degradation activity, we decided to analyze the exosome function in the presence of PaNip7 towards a poly-rAU oligo substrate that can form intra- and inter-strands base pairs, and to which PaNip7 showed high affinity for binding (Additional file 1 Figure S1). For this purpose, RNase protection assays were performed in the presence of 10 mM NaH_2_PO_4_, and samples were subjected to electrophoresis in native polyacrylamide gels. Under these conditions, it was possible to analyze both binding of the proteins to the poly-AU RNA, as well as RNA degradation by the exosome. In this experiment, we first incubated the poly-rAU with either buffer or PaNip7 for 30 minutes, after which time the exosome complexes and 10 mM NaH_2_PO_4 _were added and the reactions were further incubated for 15 minutes. The three exosome complexes bound efficiently to the poly-rAU although this RNA can form base pairs of low stability. The bands of the free RNA probe are only visible in the reactions containing low amounts of the exosome complexes (Fig. 3A; lanes 2-10), indicating that the probe is bound and consequently degraded by the exosome. This finding indicates that all exosome complexes can degrade low stability double-stranded RNAs. Despite the ability of the exosome complexes to degrade the poly-rAU oligo, it was not as effective as the poly-rA degradation (compare Figs. 2 and 3).

In the absence of PaNip7, upon addition of increasing amounts of the RNase PH ring, the free RNA band decreases in intensity, while the bands of the RNA-protein complexes, and of the degradation product increase (Fig. 3A, lanes 2-4). When incubated with RNA in the presence of PaNip7, the RNase PH ring seems to degrade only the free RNA, and not the molecules bound to PaNip7, since the free RNA band decreases in intensity while the band corresponding to the degradation product increases, and the PaNip7-RNA band remains mostly unchanged (Fig. 3A, lanes 13-15). In the cases of PaRrp4- and PaCsl4-exosome, due to the presence of the RNA binding subunits, the holo-exosome complexes bind the RNA more efficiently than the RNase PH ring (Fig. 3A, lanes 2-10). Upon incubation of PaRrp4- and PaCsl4-exosome with poly-rAU bound to PaNip7, the free RNA band decreases in intensity while exosome-RNA complexes are detected and the band of degradation products becomes visible (Fig. 3A, lanes 16-21). It is interesting to note that in all cases, much less RNA is degraded in the presence of PaNip7 (Fig. 3A, compare lanes 2-10 to lanes 13-21; Fig. 3B-D).

Although the RNase PH ring is a hexameric complex, whereas PaNip7 is a monomeric protein, the bands formed in the presence of RNase PH ring and PaNip7 run as complexes of approximately the same size. One hypothesis to explain this observation is that it is possible that more than one molecule of PaNip7 bind the same RNA, thereby forming larger RNP complexes. We ruled out the possibility that some of the PaRrp41 and PaRrp42 molecules might not be associated in the form of the RNase PH ring and bound RNA in their monomeric forms because the PaRrp41-PaRrp42 complex was purified by using size exclusion chromatography, selecting for complexes corresponding to the RNase PH ring size (Additional file 2 Figure S2). Furthermore, this complex is active for RNA degradation, confirming that the RNase PH ring was reconstituted *in vitro*.

### Pa-exosome inhibition by PaNip7 depends on its ability to bind RNA

**Figure 4:**
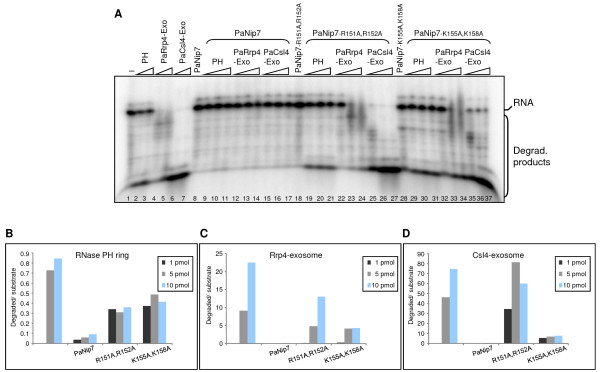
**Effect of PaNip7 mutants on the Pa-exosome poly-AU RNA degradation activity**. RNase activity reactions were performed by incubating 1 pmol of a radiolabeled 21-mer poly-rAU with 100 pmol of either PaNip7, PaNip7^R151A, R152A^, or PaNip7^K155A, K158A^, for 30 min at 37°C, after which time increasing amounts of the exosome complexes (1, 5 or 10 pmol of either RNase PH ring, PaRrp4-exosome, or PaCsl4-exosome), and NaH_2_PO_4 _(10 mM) were added to the reactions, and further incubated for 15 min at 65°C. Samples were separated on 8% denaturing polyacrylamide gels and visualized by phosphorimaging. -, No protein added to the reaction. RNA oligo and degradation products are indicated on the right-hand side.

PaNip7 shows strong inhibitory effect on exosome degradation of poly-rAU, indicating that inhibition depends, at least partially on the PaNip7 ability to bind RNA. In order to test this possibility, poly-AU RNA degradation assays were performed in the presence of PaNip7 mutants [39]. The RNase PH ring degrades poly-rAU, but not very efficiently (Fig. 4; lanes 2 and 3), whereas as the PaRrp4-exosome degrades only a few nucleotides of the poly-rAU substrate (Fig. 4; lanes 4 and 5). The PaCsl4-exosome, on the other hand, degrades completely the poly-rAU substrate (Fig. 4; lanes 6 and 7). These results are consistent with the more stable binding of PaRrp4-exosome to RNA and with the formation of a more constricted RNA entry pore in this exosome complex that does not allow double-stranded RNA molecules to be degraded. In the presence of wild type PaNip7, poly-rAU degradation was strongly inhibited (Fig. 4; lanes 9-17). Interestingly, PaNip7^R151A, R152A ^(mutant that does not bind RNA; [39]) does not inhibit RNA degradation by the exosome (Fig. 4; compare lanes 2-7 to lanes 19-27). Accordingly, the mutant PaNip7^K155A, K158A^, which binds RNA with low affinity [39], causes an intermediate inhibition (Fig. 4; lanes 29-37). These results confirm that in the case of poly-rAU, inhibition of exosome function depends on PaNip7 ability to interact with the substrate RNA. An additional control to these experiments was performed, in which the effect of Pa1135 on the RNase activity of the exosome was analyzed. Similar to PaNip7, Pa1135 binds AU-rich RNAs (Additional file 1 Figure S1), but contrary to PaNip7, Pa1135 does not inhibit the RNase activity of the exosome (data not shown), confirming the specificity of the PaNip7 effect.

### PaNip7 affects the exosome RNA polymerase activity

**Figure 5:**
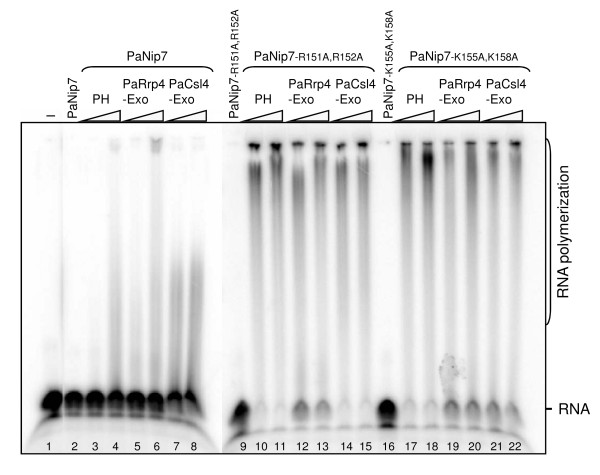
**Influence of PaNip7 and mutants on the Pa-exosome RNA polyadenylation activity**. Polyadenylation reactions were performed with a radiolabeled 14-mer poly-A RNA probe incubated with 100 pmol of PaNip7, PaNip7^R151A, R152A^, or PaNip7^K155A, K158A^, and different amounts of the exosome complexes (5 or 10 pmol of RNase PH ring, PaRrp4-exosome, or PaCsl4-exosome). Proteins were incubated with 1 pmol RNA at 37°C for 30 min, in the presence of 10 mM ADP and 10 mM MgCl_2_. Samples were separated on 8% denaturing polyacrylamide gels and visualized by phosphorimaging. -, No protein added to the reaction. RNA oligo and polymerization products are indicated on the right-hand side.

The effect of PaNip7 on the exosome RNA polymerization activity was also analyzed. Wild type PaNip7 strongly inhibited RNA polyadenylation by the exosome (Fig. 5, lanes 3-8), when compared to the PaNip7 mutants PaNip7^K155A, K158A ^and PaNip7^R151A, R152A ^(Fig. 5, lanes 10-15, and 17-22, respectively). These results further confirm that PaNip7 inhibition of the exosome activity requires an intact PaNip7, able to bind RNA. PaSBDS and Pa1135 did not affect RNA polyadenylation by the exosome complexes (data not shown), corroborating the conclusion of the specificity of the exosome inhibition by PaNip7.

### Direct interaction between PaNip7 and Pa-exosome

**Figure 6:**
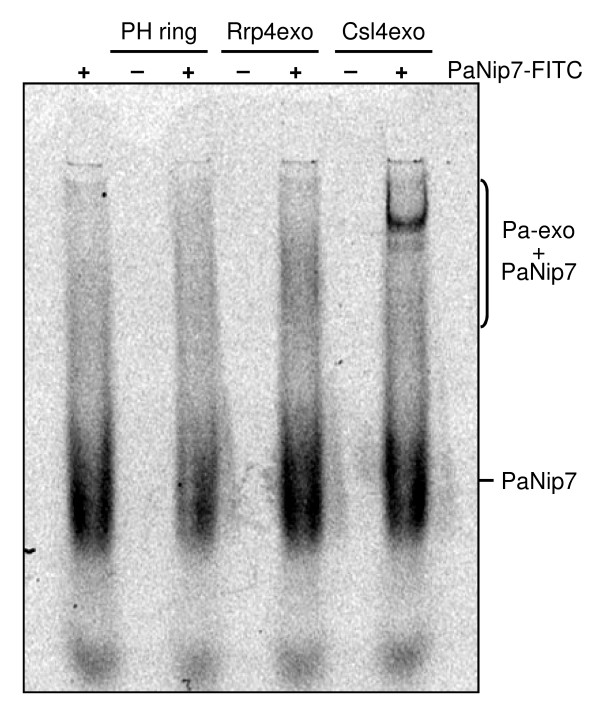
**Binding of PaNip7 to the Pa-exosome complexes**. 12.5 μg of PaNip7 labeled with FITC was incubated with 25 μg of the exosome complexes for 30 minutes at 25°C. Protein complexes were subjected to electrophoresis on 6% native polyacrylamide gels and visualized by phosphorimaging. Protein complexes are indicated on the right-hand side.

The mechanism of exosome inhibition by PaNip7 could involve competition for RNA binding. This could be the case in situations where the exosome and PaNip7 are incubated with AU-rich substrates since PaNip7 binds this type of RNA. However, PaNip7 inhibits the exosome also when the substrate is a poly-rA. Attempts to detect the interaction between PaNip7 and Pa-exosome through co-purification of the complexes using gel-filtration assays and protein pull-down experiments have failed (data not shown). This raised the hypothesis that PaNip7 might have low affinity for the Pa-exosome, or the interaction PaNip7-exosome is transient. In order to determine whether PaNip7 could interact directly with the Pa-exosome, PaNip7 was labeled with FITC (fluorescein isothiocyanate) and incubated with the exosome complexes for 30 minutes at 25°C. The resulting complexes were initially subjected to electrophoresis on native polyacrylamide gels followed by analysis of the fluorescence signal on an image plate reader. The results from these assays show that PaNip7 binds to the holo-exosome complexes, with a higher affinity for the PaCsl4-exosome than for the PaRrp4-exosome (Fig. 6). These data corroborate the stronger inhibitory effect of PaNip7 upon PaCsl4-exosome described above.

**Figure 7:**
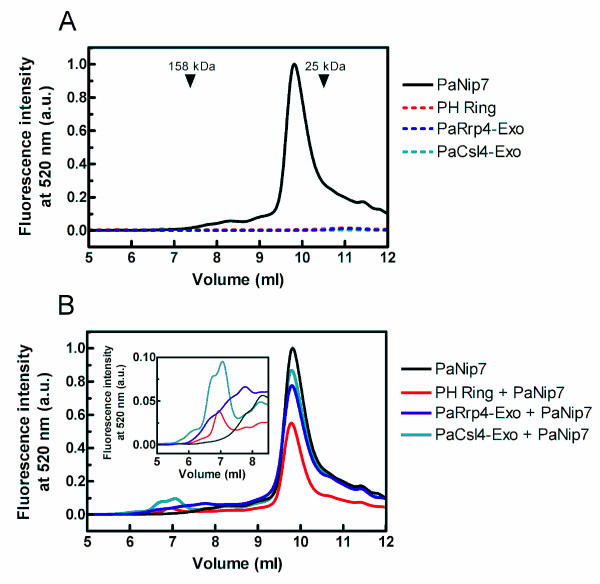
**Binding of PaNip7 to the Pa-exosome complexes analyzed by size exclusion chromatography**. 12.5 μg of PaNip7 labeled with FITC was incubated with 25 μg of the exosome complexes for 30 minutes at 25°C. Protein complexes were subjected to size exclusion chromatography. Fluorescence emission was detected at 520 nm. (**A**) separate proteins. (**B**) PaNip7 incubated with the exosome complexes. Molecular mass standards used were aldolase (158 kDa) and chymotrypsin (25 kDa).

FITC-labeled PaNip7 incubated with the exosome complexes as described above was also analyzed by size exclusion chromatography. The analysis of fluorescence emission from the isolated proteins show that most PaNip7 is eluted in fractions corresponding to proteins of about 30 kDa, while the exosome complexes do not show fluorescence emission at 520 nm (Fig. 7A). When the exosome complexes are incubated with FITC-labeled PaNip7, however, fluorescence in the position corresponding to the exosome-PaNip7 complexes can be detected (Fig. 7B). The signal is stronger for PaCsl4-exosome + PaNip7, indicating a more stable interaction between these proteins. These results further confirm that PaNip7 inhibits the exosome by associating with both the complex and the substrate RNA.

## Discussion

The archaeal exosome has been shown to bind, degrade and polyadenylate RNA *in vitro *[18-22]. Recently, the *S. solfataricus *exosome activities were analyzed *in vitro *with respect to the different subunit composition, Mg^2+ ^concentration, and the efficiency of polymerization and degradation of RNA, which depend on the concentration of free phosphate or nucleotide diphosphates [41]. We have previously compared the RNA binding abilities between the *Pyrococcus horikoshii *apo-exosome RNase PH ring and the *P. horikoshii-abysii *Rrp4-exosome complex [21]. Here we extended the comparison of the RNA binding abilities, and RNA degradation and polymerization activities of three different *P. abyssi *exosome complexes, RNase PH ring, PaRrp4-exosome and PaCsl4-exosome. Furthermore, since very little is known of the possible archaeal exosome regulatory factors, here we analyzed the effects of three proteins on the exosome activities when degrading different substrates.

The nine-subunit PaRrp4-exosome binds RNA with higher affinity but degrades RNA less efficiently than PaCsl4-exosome. These different characteristics could be explained by PaRrp4 having higher affinity for RNA than PaCsl4, due to the presence of one S1 and one KH domain in PaRrp4, instead of one S1 and one zinc-ribbon domain in PaCsl4 [4,18]. Consequently, depending on the RNA binding subunit present in the complex, the structure of the exosome RNA entry pore may undergo conformational changes [18], leading to the different RNA binding affinities of the exosome complexes and the slower RNA degradation by the PaRrp4-exosome. Although the archaeal exosome binds the substrate RNA directly, it may also interact with various protein factors that are important for directing the complex to the substrate, for opening RNA secondary structures, and possibly for regulating its function.

The bacterial PNPase, which is structurally and functionally related to the exosome, is part of the degradosome, a protein complex involved in rRNA and tRNA processing and in mRNA degradation that is formed by PNPase, RNase E, the helicase RhlB, and enolase [reviewed in [42]]. In eukaryotes, exosome co-factors include the helicase Mtr4p, the TRAMP complex, Nop53p, Dob1p, Rrp47p, Npl3p, Lsm proteins, the Ski complex, and the Nrd1p/Nab3p complex [24,26-28,43]. Yeast Nip7p has been shown to interact with the exosome subunit Rrp43p [40]. Conditional depletion of yeast Nip7p leads to the accumulation of pre-rRNA 27S, a precursor of 5.8S and 25S rRNAs [40]. Nip7p interacts with RNA *in vitro *and with several proteins known to associate with the pre-rRNA 27S [36,40,44]. The yeast and archaeal orthologues of Nip7 contain a PUA domain, which has been previously demonstrated to be involved in RNA interaction [39,45]. Accordingly, Nip7p and PaNip7 bind RNA *in vitro*, with higher affinity for poly-U RNAs [39]. Here we show that PaNip7 also has high affinity for poly-AU RNAs that can form weak secondary structures. PaNip7 strongly inhibits the archaeal exosome, and this inhibition depends on PaNip7 ability to bind RNA and to interact with the exosome complex. Supporting this conclusion, PaNip7 mutants that do not bind RNA, have smaller inhibitory effects on the exosome. It is possible that PaNip7 binds RNA through its C-terminal PUA domain, and interacts with the archaeal exosome via its N-terminal domain, thereby controlling the exosome function. The hypothesis that the inhibition of the exosome by PaNip7 involves both RNA binding and protein interaction is further strengthened by the observations that PaNip7 also inhibits the degradation of RNAs for which PaNip7 has low affinity, such as poly-rA. Interestingly, PaNip7 has stronger inhibitory effect on PaCsl4-exosome, indicating a possible role for PaNip7 as a regulatory factor for one of the exosome complexes. PaNip7 could interact with PaCsl4 Zn-ribbon domain, which may be involved in the interaction with proteins, providing a mechanism for the exosome regulation.

Pa1135 was shown here to bind poly-rAU RNA but contrary to PaNip7 does not strongly affect the exosome RNase activity, although Pa1135 inhibits RNA binding by the RNase PH ring and PaCsl4-exosome complexes. The *P. horikoshii *RNase P is formed by the RNA component and five proteins, including Rrp30 [46]. Since Pa1135 is encoded by a gene found in the same operon as the RNase P subunit Rrp30 [4], it is possible that Pa1135 regulates RNase P function *in vivo*.

Although eukaryotic SBDS/Sdo1p has not been shown to interact with the exosome, it has been shown to be required for pre-rRNA processing, for 60S ribosomal subunit translational activation and Tif6p recycling [35]. In addition, SBDS has been shown to interact with the human orthologue of Nip7p and its deficiency affects expression of different genes [36]. Yeast Sdo1p has recently been shown to interact with Nip7p and to bind poly-A and poly-AU RNA [38]. The PaSBDS gene is found in the same operon as three of the exosome subunits, indicating that it is also involved in RNA metabolism.

Structural analysis of *Archeoglobus fulgidus *SBDS has shown that this protein contains an RNA binding domain [47]. In this work, we confirmed the hypothesis of PaSBDS binding to RNA, by showing that it binds poly-rA, poly-rU and poly-rAU *in vitro *in a length-dependent manner, and competes with the PaRrp4-exosome for binding to A-rich RNAs. These results indicate that PaSBDS is also involved in RNA processing and may regulate one of the archaeal exosome complexes *in vivo*. Further indication of exosome regulation was obtained in RNA degradation assays, in which the RNase activity of the RNase PH ring and of the PaRrp4-exosome was slightly inhibited by PaSBDS. It is therefore possible that PaSBDS interacts with RNA *in vivo *and controls its processing by the exosome.

## Conclusions

We show in this work that two archaeal proteins that bind RNA can affect the Pa-exosome activity, making them candidates to be exosome regulatory factors. PaSBDS binds A- and AU-rich RNAs and inhibits mainly the PaRrp4-exosome. PaNip7 binds preferably AU-rich RNAs and strongly inhibits PaCsl4-exosome. Furthermore, similar to the eukaryotic counterparts, PaNip7 interacts with the Pa-exosome. Based on the results shown here that PaSBDS and PaNip7 inhibit preferentially specific exosome complexes, it is possible that these proteins control the Pa-exosome *in vivo*. The evolutionary conserved structures and RNA affinities of Nip7 and SBDS raise the hypothesis that their eukaryotic orthologues also control the exosome function.

## Methods

### Microorganisms, Plasmids, Enzymes, and DNA Manipulation

The *Escherichia coli *strains used in this study were DH5α and BL21-CodonPlus (DE3)-RIL (Stratagene). Genomic DNA of *P. abyssi *GE5 was kindly provided by Dr. Patrick Forterre (Institut de Génétique et Microbiologie, Université Paris Sud, France). Plasmid DNA was extracted using Qiagen plasmid purification systems. Restriction enzymes and other DNA-modifying enzymes were used as recommended by the manufacturer (New England Biolabs).

### Construction of Expression Vectors

Plasmids for *E. coli *expression of PaRrp4 [21], wild type PaNip7 [48] and mutants PaNip7^R151A, R152A ^and PaNip7^K155A, K158A ^[39] have been described previously. PaSBDS (PAB0418) was PCR amplified from *P. abyssi *genomic DNA using primers PaSBDSfor (5'-AGGATCCCATATGCCTATTAGCGTTG-3') and PaSBDSrev (5'-CGGCCTCGAGTCATAGCCCCTT-3'), digested with *BamH*I-*Xho*I and inserted into vector pET-28a (Novagen), also digested with *BamH*I-*Xho*I. Pa1135 coding sequence was PCR-amplified from *P. abyssi *genomic DNA using primers Pa1135for (5'-GTTAGGGGGGATCCATGGCAG-3') and Pa1135rev (5'-CGGCCTCGAGTCAATCCTCCC-3'), and inserted into vector pET28a, digested with *BamH*I-*Xho*I. The PaRRP41 coding sequence was amplified from *P. abyssi *genomic DNA using primers PaRRP41for (5'-TACTCGAGCATATGATGGAGAAACCAGAAG-3') and PaRRP41rev (5'-ACGAATTCATCTCATTATCACTCACTTTC-3') and inserted into the PCR product cloning vector pGEM-T (Promega) prior to subcloning into the *Nde*I and *Sal*I restriction sites of the expression vector pET29a (Novagen). PaRRP42 was amplified using primers PaRRP42for (5'-AGGGATCCCATATGAGTGATAATGAGATCG-3') and PaRRP42rev (5'-TACGCGTATCGATGTTATATCATTGCTTTGC-3'), inserted into pGEM-T vector (Promega) and subsequently subcloned into the *BamH*I and *EcoR*I sites of the expression vector pAE [49]. PaCSL4 was amplified using primers PaCSL4for (5'-AGATCTCATATGGAGGAAGGTGAGGAG-3') and PaCSL4rev (5'-AGAATTCTTTGCCCTCATAGCTTC-3') and inserted into the *Nde*I and *EcoR*I sites of the expression vector pET28a (Novagen).

### Expression and Purification of Proteins

Recombinant proteins were expressed in the *E. coli *BL21-CodonPlus (DE3)-RIL strain, transformed with the correspondent plasmids. Expression was induced by addition of 10 mM lactose or, in the case of PaSBDS, 0.5 mM isopropyl 1-thio-β-D-galactopyranoside (IPTG). Cells expressing PaRrp41 and PaRrp42 were harvested and suspended in buffer A (30 mM Tris-HCl, pH 8.0, 150 mM NaCl, 5 mM imidazole). Cells expressing PaSBDS were suspended in buffer B (30 mM Tris-HCl, pH 7.0, 500 mM NaCl, 5 mM imidazole). Cells expressing Pa1135 were suspended in buffer C (30 mM Tris-HCl, pH 8.0, 500 mM NaCl, 5 mM imidazole). All cells were lysed in a French press. The lysate was heated at 85°C for 30 min and cooled on ice for 15 min. After centrifugation at 20,000 × *g *for 30 min, the supernatant was fractionated by affinity chromatography in Ni-NTA-agarose (Qiagen), followed by gel filtration in superdex 75 (GE Healthcare).

PaNip7, PaNip7^R151A, R152A ^and PaNip7^K155A, K158A ^were purified as described previously [39,48]. To reconstitute the archaeal exosome, the RNase PH ring complex was obtained by co-expressing the proteins PaRrp41 and PaRrp42 in *E. coli*, transformed with the plasmids pET29-PaRrp41 (Kan^R^) and pAE-PaRrp42 (Amp^R^). For PaRrp4-exo assembly, cells expressing PaRrp4 were mixed with cells expressing PaRrp41 and PaRrp42, prior to lysis. PaCsl4 was obtained by co-expression with PaRrp42. PaCsl4+PaRrp42-expressing cells were mixed to PaRrp41+PaRrp42-expressing cells to obtain the PaCsl4-exo. Protein samples were concentrated by centrifugation using Centricon microconcentrators (Millipore). Protein content was determined by bicinchoninic acid (BCA) assay for protein quantitation (Sigma).

### RNase PH Phosphorolysis and Polymerase Assays

RNA phosphorolysis was assayed by incubating the complexes with 1 pmol ^32^P-labeled RNAs in Tris-50 buffer (10 mM Tris-HCl, pH 8.0, 50 mM KCl, 10 mM MgCl_2_, 1 mM DTT, 100 μg/ml BSA), and 10 mM NaH_2_PO_4_, in 10 μl at 65°C for 15 minutes. RNA polymerization was accomplished under the same conditions, but using ADP instead of NaH_2_PO_4_, in 20 μl at 37°C for 30 minutes. RNA degradation and polymerization products were resolved on 8% or 10% denaturing polyacrylamide gel and visualized on a Phosphorimager (MolecularDynamics).

### RNA binding assay

RNA binding assays were carried out with 1 pmol ^32^P 5'-labeled 14-mer poly-rA, poly-rU, 13-mer poly-rC, and 21-mer poly-rAU (5'-UUAUUAUUUAUUUAUUAUUUA-3') oligoribonucleotides (IDT). The assays were performed under the same conditions as described previously [21] or in 20 mM Tris-HCl pH 8.0, 20 mM KCl, 2 mM MgCl_2 _1 mM DTT, 100 μg/ml BSA, 0.8 U RNasin. Different concentrations of proteins were incubated with the substrate RNA in 20 μl at 37°C for 30 minutes. The samples were resolved on 8% native polyacrylamide gels and visualized on a Phosphorimager (MolecularDynamics).

### RNA protection assays

RNA protection assays were carried out with 1 pmol ^32^P 5'-labeled poly(rAU) oligoribonucleotides. Protein cofactors were incubated with the RNAs for 30 min at 37°C, after which time the exosome complexes and NaH_2_PO_4 _(to the final concentration of 10 mM) were added to the reactions, which were incubated at 65°C for further 15 min. The products were resolved on native or denaturing 8% polyacrylamide gels and visualized on a Phosphorimager (MolecularDynamics).

### Quantitative analysis of exosome activities in vitro

A Phosphorimager (MolecularDynamics) was used for quantitation of the bands obtained from RNA degradation, polyadenylation and binding assays.

### Protein interaction assays

PaNip7 was labeled with FITC (fluorescein isothiocyanate), by following the instructions of the manufacturer's protocol (Molecular Probes). 12.5 μg of PaNip7-FITC were incubated with 25 μg of the exosome complexes at 25°C for 30 minutes. The samples were subsequently subjected to electrophoresis on native 6% polyacrylamide gels, and the fluorescence was analyzed on a Phosphorimager (MolecularDynamics). Concomitantly, samples were subjected to size exclusion chromatography and the absorbance was monitored at 280 nm and the fluorescence was monitored at 520 nm.

## Authors' contributions

JSL purified the recombinant proteins and carried out the activity assays, protein interaction assays and drafted portions of the manuscript. CRRR cloned the archaea exosome genes, stablished the protein purification protocols, and drafted portions of the manuscript. MCTS helped with the purification of proteins. PPC cloned PaNip7 gene and performed mutagenesis to obtain PaNip7 mutants. FLP helped with PaNip7 labeling and analysis of protein complexes. NITZ and DF coordinated parts of the work, participated in the interpretation of data and drafted portions of the manuscript. CCO designed, organized and coordinated the experiments, drafted the manuscript and edited the final text. All authors read and approved the final manuscript.

## Supplementary Material

Additional file 1 **Figure S1: Analysis of PaSBDS, PaNip7 and Pa1135 interaction with RNA oligonucleotides *in vitro***. Electrophoretic mobility shift assays with different radiolabeled RNA probes incubated with the indicated amounts of purified proteins. Proteins were incubated with 1 pmol of 10-mer, 12-mer or 14-mer poly-rA, 13-mer poly-rC, 14-mer poly-rU, or 21-mer poly-rAU RNA oligos at 37°C for 30 min. RNA-protein complexes were fractionated on 8% native polyacrylamide gels and visualized by phosphorimaging. (**A**) Increasing amounts of PaSBDS were added to the reactions: 50, 100, 200 or 400 pmol. PaSBDS binds poly-rA, poly-rU and poly-rAU. (**B**) 10, 50 or 100 pmol of PaNip7 were incubated with poly-rC or poly-rAU. PaNip7 binds poly-rAU efficiently. (**C**) 50, 100, 200 or 400 pmol of Pa1135 were incubated with poly-rA, poly-rU, poly-rC or poly-rAU. Pa1135 binds poly-rAU efficiently. -, No protein was added to the reaction. Free structured and unstructured RNA oligos and protein-RNA complexes are indicated on the right hand side. Click here for file

Additional file 2 **Figure S2: Purification of Pa-exosome complexes and regulatory proteins**. Coomassie-stained polyacrylamide gels showing proteins purified through size exclusion chromatography. (**A**) Purification of RNase PH ring. (**B**) Purification of PaRrp4-exosome. (**C**) Purification of PaCsl4-exosome. (**D**) Purification of PaNip7. (**E**) Purification of PaSBDS. (**F**) Purification of Pa1135. (**G**) Purification of PaNip7R151A, R152A. (**H**) Purification of PaNip7K155A, K158A. M, molecular weight marker; TE, total extracts; FT, flow through; W, wash. Click here for file
