# Supplementary material for: Identification of archaeal proteins that affect the exosome function in vitro
Source: BMC Biochem. 2010 May 27;11:22. doi: 10.1186/1471-2091-11-22 (PMC2890523; doi:10.1186/1471-2091-11-22)
Supplement: Additional file 1 — Figure S1: Analysis of PaSBDS, PaNip7 and Pa1135 interaction with RNA oligonucleotides in vitro. Electrophoretic mobility shift assays with different radiolabeled RNA probes incubated with the indicated amounts of purified proteins. Proteins were incubated with 1 pmol of 10-mer, 12-mer or 14-mer poly-rA, 13-mer poly-rC, 14-mer poly-rU, or 21-mer poly-rAU RNA oligos at 37°C for 30 min. RNA-protein complexes were fractionated on 8% native polyacrylamide gels and visualized by phosphorimaging. (A) Increasing amounts of PaSBDS were added to the reactions: 50, 100, 200 or 400 pmol. PaSBDS binds poly-rA, poly-rU and poly-rAU. (B) 10, 50 or 100 pmol of PaNip7 were incubated with poly-rC or poly-rAU. PaNip7 binds poly-rAU efficiently. (C) 50, 100, 200 or 400 pmol of Pa1135 were incubated with poly-rA, poly-rU, poly-rC or poly-rAU. Pa1135 binds poly-rAU efficiently. -, No protein was added to the reaction. Free structured and unstructured RNA oligos and protein-RNA complexes are indicated on the right hand side. [file 1471-2091-11-22-S1.PDF]

Additional File 1

Figure S1. Analysis of PaSBDS, PaNip7 and Pa1135 interaction with RNA oligonucleotides *in vitro*. Electrophoretic mobility shift assays with different radiolabeled RNA probes incubated with the indicated amounts of purified proteins. Proteins were incubated with 1 pmol of 10-mer, 12-mer or 14-mer poly-rA, 13-mer poly-rC, 14-mer poly-rU, or 21-mer poly-rAU RNA oligos at 37°C for 30 min. RNA-protein complexes were fractionated on 8% native polyacrylamide gels and visualized by phosphorimaging. (**A**) Increasing amounts of PaSBDS were added to the reactions: 50, 100, 200 or 400 pmol. PaSBDS binds poly-rA, poly-rU and poly-rAU. (**B**) 10, 50 or 100 pmol of PaNip7 were incubated with poly-rC or poly-rAU. PaNip7 binds poly-rAU efficiently. (**C**) 50, 100, 200 or 400 pmol of Pa1135 were incubated with poly-rA, poly-rU, poly-rC or poly-rAU. Pa1135 binds poly-rAU efficiently. –, No protein was added to the reaction. Free structured and unstructured RNA oligos and protein-RNA complexes are indicated on the right hand side.

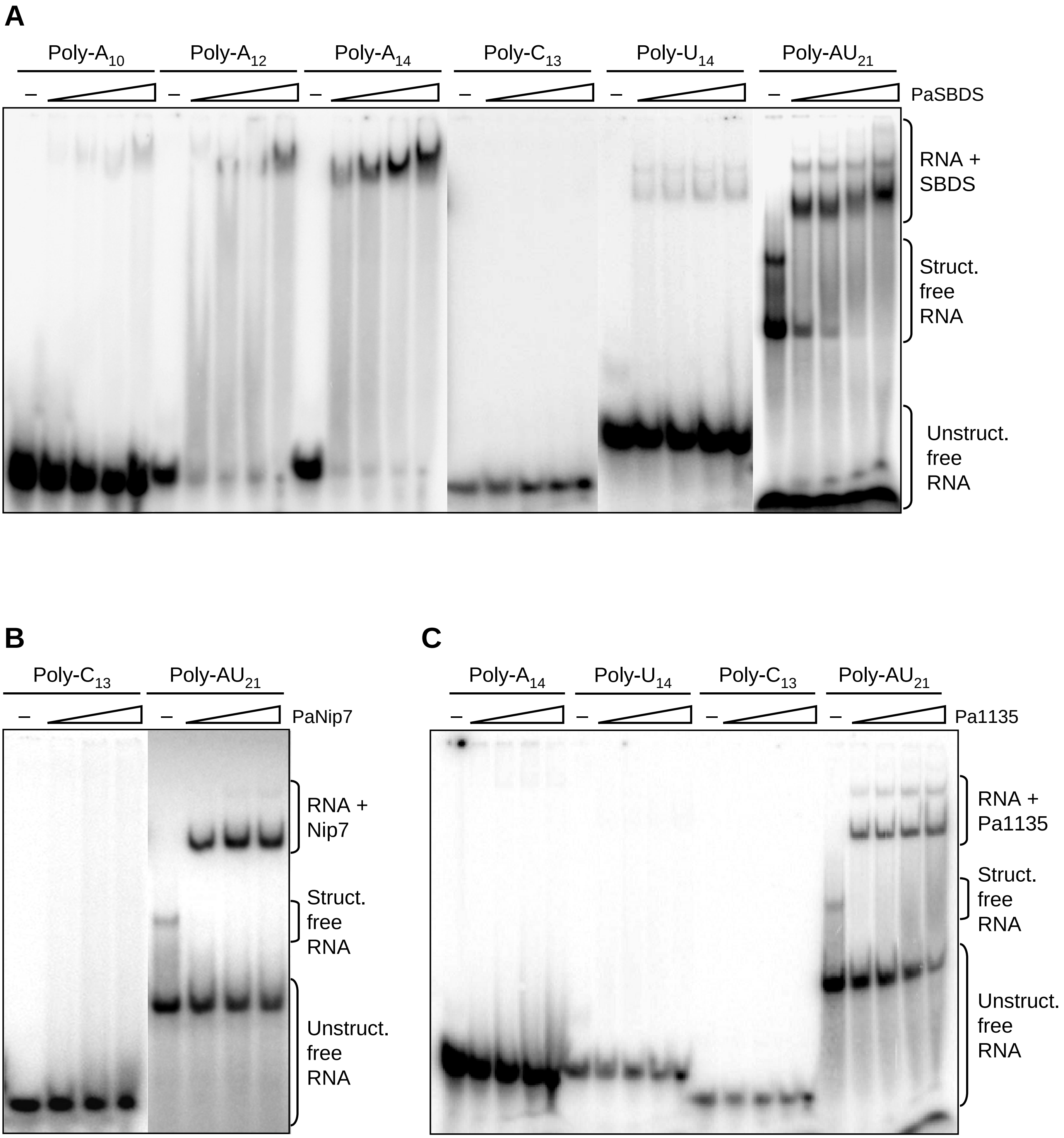

Figure S1 Luz et al.
