# Supplementary material for: Identification of archaeal proteins that affect the exosome function in vitro
Source: BMC Biochem. 2010 May 27;11:22. doi: 10.1186/1471-2091-11-22 (PMC2890523; doi:10.1186/1471-2091-11-22)
Supplement: Additional file 2 — Figure S2: Purification of Pa-exosome complexes and regulatory proteins. Coomassie-stained polyacrylamide gels showing proteins purified through size exclusion chromatography. (A) Purification of RNase PH ring. (B) Purification of PaRrp4-exosome. (C) Purification of PaCsl4-exosome. (D) Purification of PaNip7. (E) Purification of PaSBDS. (F) Purification of Pa1135. (G) Purification of PaNip7R151A, R152A. (H) Purification of PaNip7K155A, K158A. M, molecular weight marker; TE, total extracts; FT, flow through; W, wash. [file 1471-2091-11-22-S2.PDF]

Additional File 2

Figure S2. Purification of Pa-exosome complexes and regulatory proteins. Coomassie-stained polyacrylamide gels showing proteins purified through size exclusion chromatography. (**A**) Purification of RNase PH ring. (**B**) Purification of PaRrp4-exosome. (**C**) Purification of PaCsl4-exosome. (**D**) Purification of PaNip7. (**E**) Purification of PaSBDS. (**F**) Purification of Pa1135. (**G**) Purification of PaNip7^{R151A,R152A}. (**H**) Purification of PaNip7^{K155A,K158A}. M, molecular weight marker; TE, total extracts; FT, flow through; W, wash.

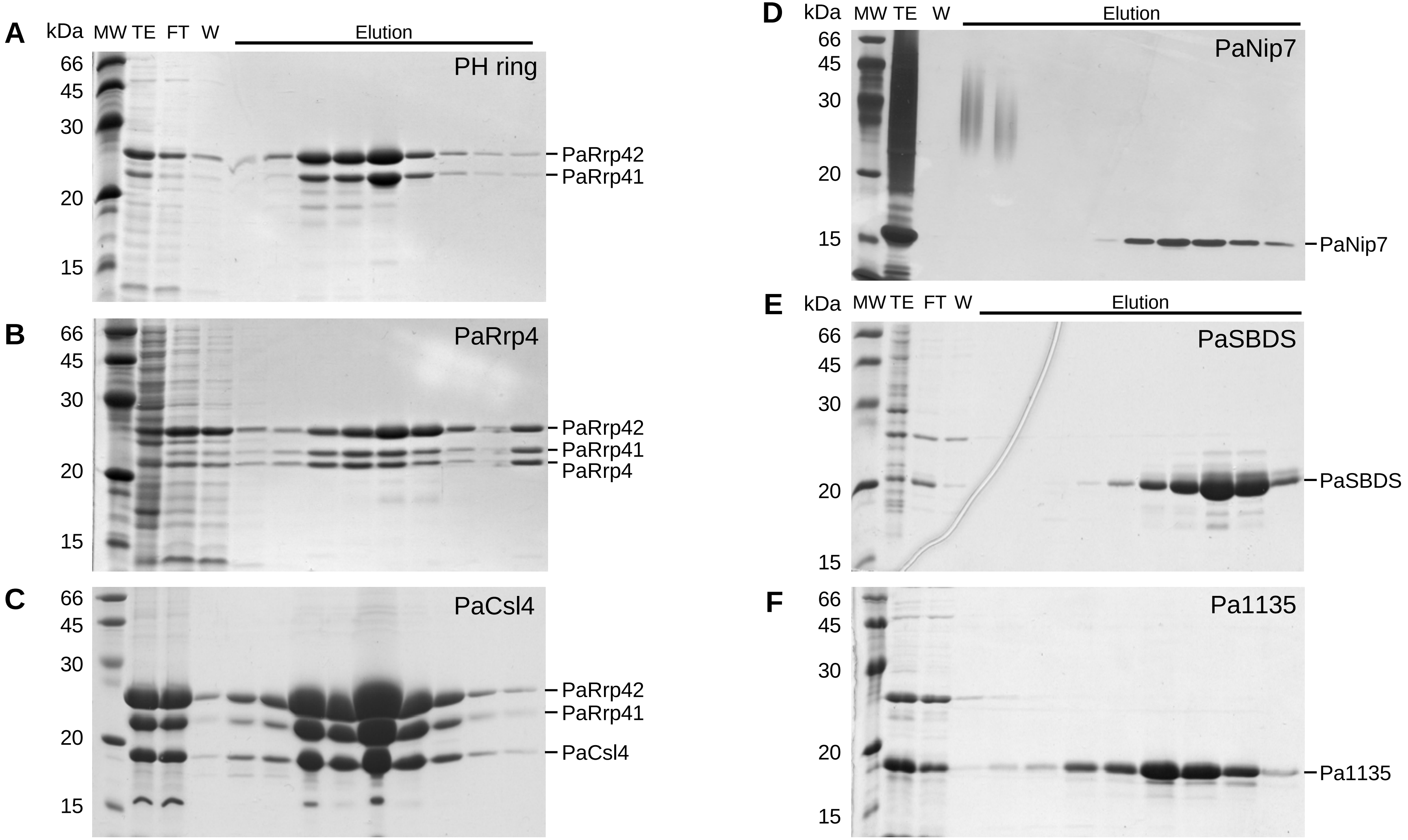

Figure S2 Luz et al.
